# Supplementary material for: The Perspective of Arctic–Alpine Species in Southernmost Localities: The Example of Kalmia procumbens in the Pyrenees and Carpathians
Source: Plants (Basel). 2023 Sep 26;12(19):3399. doi: 10.3390/plants12193399 (PMC10574852; doi:10.3390/plants12193399)
Supplement: Supplementary file 1 [file plants-12-03399-s001.zip › plants-2576877-supplementary.pdf]

The perspective of Arctic-Alpine species in southernmost localities: *Kalmia procumbens* example in the Pyrenees and Carpathians

Walas Ł., et al.

Supplementary materials

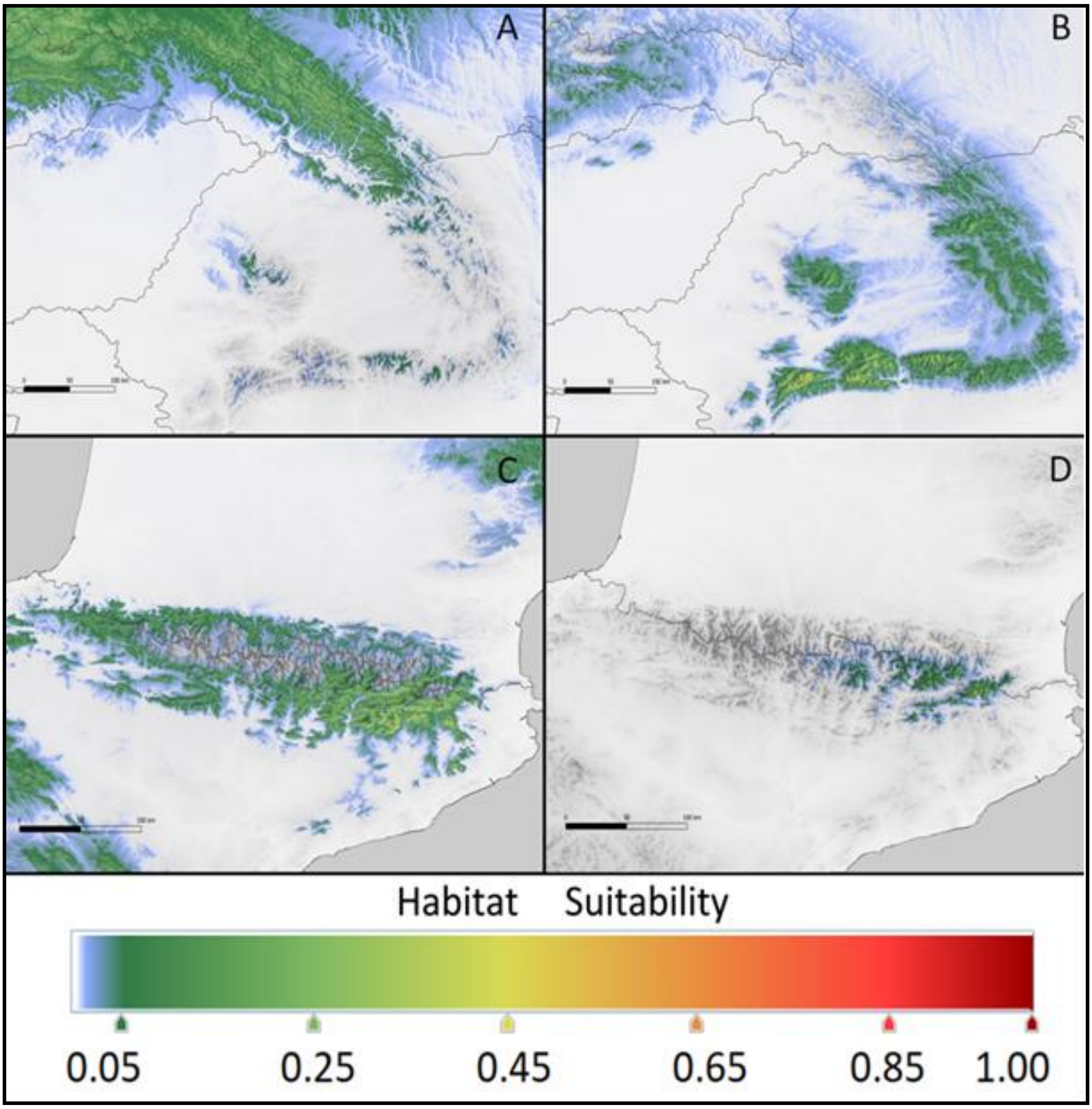

Figure S1. Provided potential range of *Kalmia procumbens* during Last Glacial Maximum in the Carpathians (a and b) estimated using: a – environmental conditions from the East Carpathians, b – from the South Carpathians; in the Pyrenees (c and d) using: c – conditions from the East Pyrenees, d – from the Central Pyrenees.

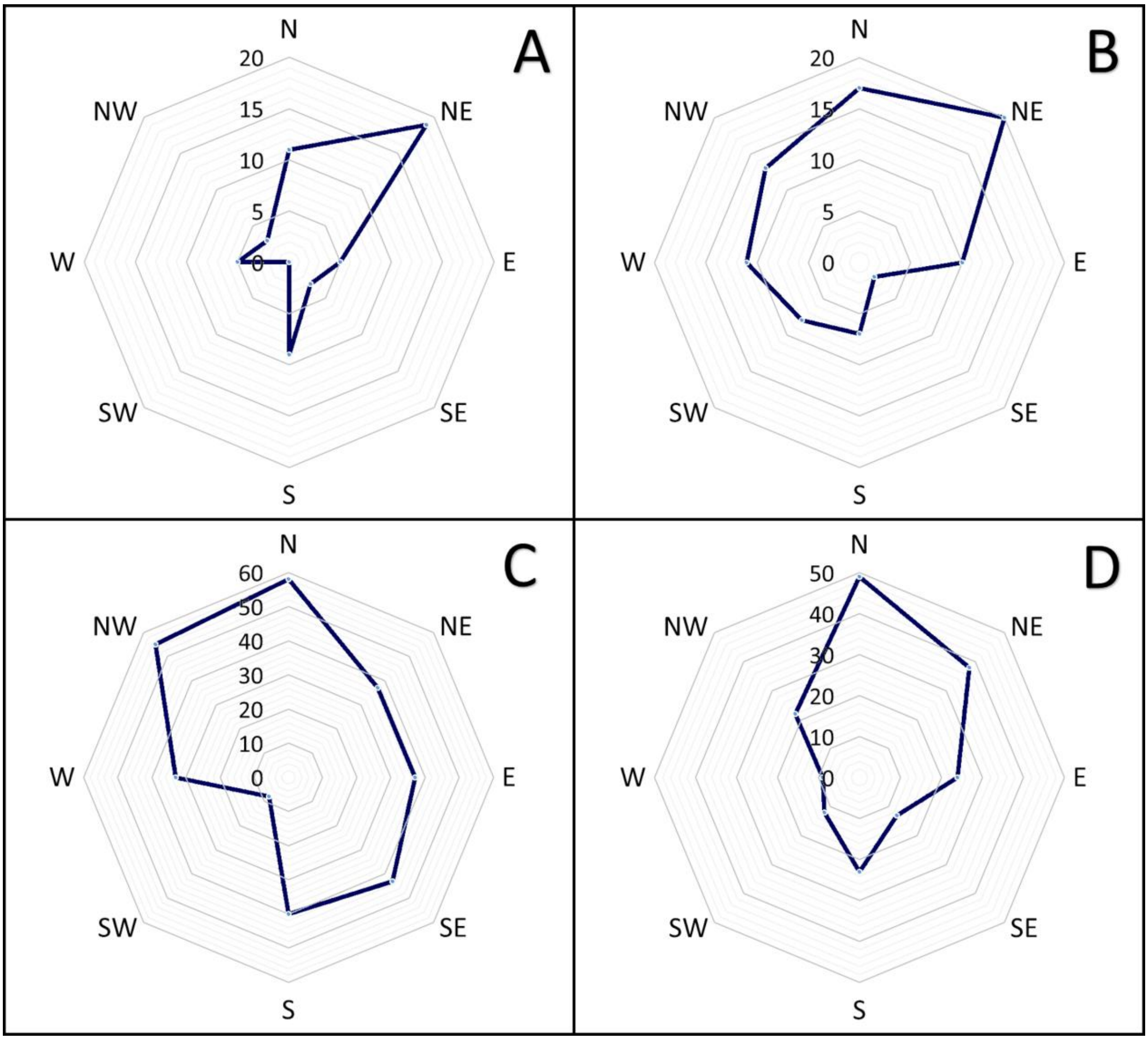

Figure S2. Occurene of populations of *Kalmia procumbens* in different exposition in the Carpathians (a and b): a – East Carpathians, b –South Carpathians; and in the Pyrenees (c and d): c –East Pyrenees, d –Central Pyrenees.

Table S1. Altitudinal maxima of subalpine and alpine species in the Carpathians [48,103,104] and Pyrenees [28]; CEUR – Central European mountain, ARALP – Arctic-Alpine; EUROS – Euro-Siberian mountain

Species	General range	Carpathias	Pyrenees
Salix reticulata	ARALP	2500	3070
Dryas octopetala L.	ARALP	2150	3040
Juniperus communis L. var. *saxatilis* Pall. (=*Juniperus communis* subsp. *alpina* (Sm.) Čelak., *J. commnis* subsp. *nana* (Baumg.) Syme)	ARALP	2350	3080
Salix bicolor Ehrh. ex Willd.	CEUR	1860	3070
Salix hastata L.	ARALP	2150	2400
Salix herbacea L.	ARALP	2600	3080
Vaccinium gaultherioides Bigelow	ARALP	2510	3000
